# Is *Culex modestus* a New Usutu virus vector?

**DOI:** 10.1186/s13071-024-06360-z

**Published:** 2024-07-02

**Authors:** Alina Soto, Lotte Wauters, Leen Delang

**Affiliations:** Department of Microbiology, Immunology and Transplantation, Virus-Host Interactions & Therapeutic Approaches (VITA) Research Group, Rega Institute for Medical Research, KU Leuven, Leuven, Belgium

**Keywords:** Usutu virus, *Culex modestus*, Vector competence, Arbovirus, Emerging pathogen

## Abstract

Usutu virus is an emerging pathogen transmitted by mosquitoes. *Culex modestus* mosquitoes are widespread in Europe, but their role in disease transmission is poorly understood. Recent data from a single infectious mosquito suggested that *Culex modestus* could be an unrecognized vector for Usutu virus. In this study, our aim was to corroborate this finding using a larger sample size. We collected immature *Culex modestus* from a reedbed pond in Flemish Brabant, Belgium, and reared them in the laboratory until the third generation. Adult females were then experimentally infected with Usutu virus in a blood meal and incubated at 25 °C for 14 days. The presence of Usutu virus in the saliva, head and body of each female was determined by plaque assay and quantitative real-time reverse-transcription polymerase chain reaction (qRT-PCR). The transmission efficiency was 54% (*n* = 15/28), confirming that Belgian *Culex modestus* can experimentally transmit Usutu virus.

**Graphical Abstract:**

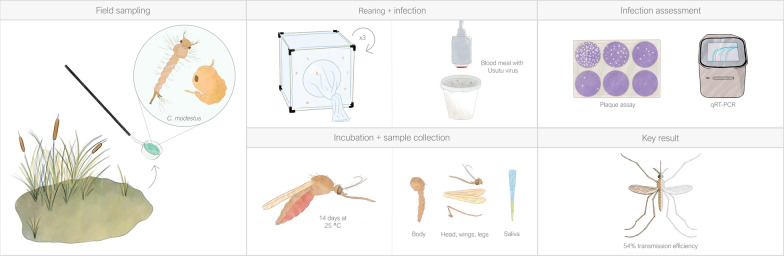

## Brief report

Usutu virus (USUV) is an emerging arthropod-borne virus endemic to Europe [1]. The virus cycles between avian reservoir hosts and intermediate mosquito vectors. USUV can spread rapidly, causing systemic disease and mass mortality in many bird species [2]. Humans can become incidental or dead-end hosts of USUV when bitten by an infected mosquito. The primary USUV vector is considered to be the Northern house mosquito *Culex pipiens* sensu lato (s.l.) [1]. In a recent study with field-collected *Culex* mosquitoes from Belgium, we observed that a *Culex modestus* mosquito could transmit USUV (20% transmission efficiency; *n* = 1/5), whereas the *Culex pipiens* form *pipiens* from the same study could not (0% transmission efficiency; *n* = 0/37) [3]. Whilst *Culex pipiens* s.l. have been shown to experimentally transmit USUV [4], we hypothesize that, in addition to *Culex pipiens*, *Culex modestus* is an important but so far unrecognized USUV vector. To follow up on our previous study, our aim was to corroborate these preliminary findings using a larger sample of *Culex modestus*.

We captured adult mosquitoes from July to August 2023 at a known *Culex modestus* habitat in Leuven, Belgium (Arenberg Park, N 50°51′46, E 4°41′01), as described in a previous article [3]. We were unable to capture sufficient mosquitoes for an experimental infection, as only 2.2% of adult mosquitoes captured over 25 trap nights were female *Culex modestus* (*n* = 2/91). We therefore opted to capture immature mosquitoes from a reedbed pond at the same location using dippers (John W. Hock Company, Gainesville, FL, USA). A total of 675 larvae and pupae were captured at Arenberg Park from August to October 2023. The immature mosquitoes were transported to the insectary facility and reared at 25 °C in plastic containers containing 500 ml tap water. Sprinkles of fish food (Tetramin^®^, Tetra, Spectrum Brands Pet, LLC, Blacksburg, USA) were provided daily until adult emergence. Of the surviving mosquitoes that emerged into adults, a total of 12 female and 10 male *Culex modestus* (*n* = 22/150) were identified using the key of Becker [5]. These mosquitoes were placed in 32.5 cm^3^ BugDorm cages (MegaView Science Co., Ltd., Taichung, Taiwan) at 25 °C and 70% relative humidity (RH) with access to 10% sucrose ad libitum on cotton pledgets. The females were given bowls containing water from their original breeding site to lay autogenous egg rafts, which were used to produce a subsequent generation. The larvae of *Culex modestus* were reared in breeding site water with sprinkles of fish food. This process was repeated until the third generation (F_3_) was reached. The F_3_ females were given 7–14 days to lay their autogenous egg rafts before offering them an infectious bloodmeal.

F_3_ female *Culex modestus* were taken to a biosafety-level 3 facility to determine their vector competence as described previously [3], with minor adaptations. Adult females of 7–14 days old were offered an infectious bloodmeal consisting of washed rabbit blood re-suspended with foetal bovine serum (FBS), 5 mM adenosine triphosphate (ATP) and 1.0 × 10^7^ 50% tissue culture infectious dose (TCID_50_)/ml USUV African lineage 3 (Grivegnée strain, passage 6, collected in Belgium [6]). A 69% feeding rate was observed (*n* = 44/64). The fed females were incubated at 25 °C and 70% RH for 14 days, after which saliva was collected from 28 surviving females. Individual mosquito bodies or the heads, wings and legs (combined) were placed in homogenate tubes containing 300 µl PBS and 2.8 mm Precellys ceramic beads (Bertin Technologies, Montigny-le-Bretonneux, France). These samples were processed by homogenization (6800 rpm for 1 min) and filtration through a 0.8 µM filter. USUV infection was assessed as described previously by plaque assay on baby hamster kidney (BHK) cells [3]. All samples were incubated for 3 days to measure plaque forming units (PFU) per sample. RNA from the body and the head, wings and legs of each mosquito was extracted and quantified by qRT-PCR as described previously [3]. All figures were generated using GraphPad Prism v10.1.1 (GraphPad Software, San Diego, California USA), and the illustrations in the graphical abstract were made using Procreate.com.

**Fig. 1:**
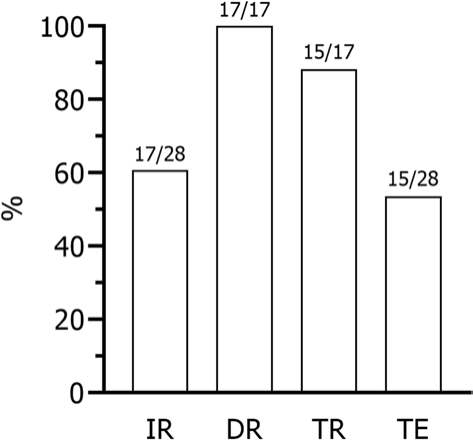
The vector competence of Belgian *Culex modestus* for USUV. The proportion of USUV positivity (%) was determined by both plaque assay and qRT-PCR. *IR* infection rate (*n* with positive body/*n* total), *DR* dissemination rate (*n* with positive head, wings and legs/*n* with positive body), *TR* transmission rate (*n* with positive saliva/*n* with positive head, wings and legs), *TE* transmission efficiency (*n* with positive saliva/*n* total). Sample size is indicated above each bar

**Fig. 2:**
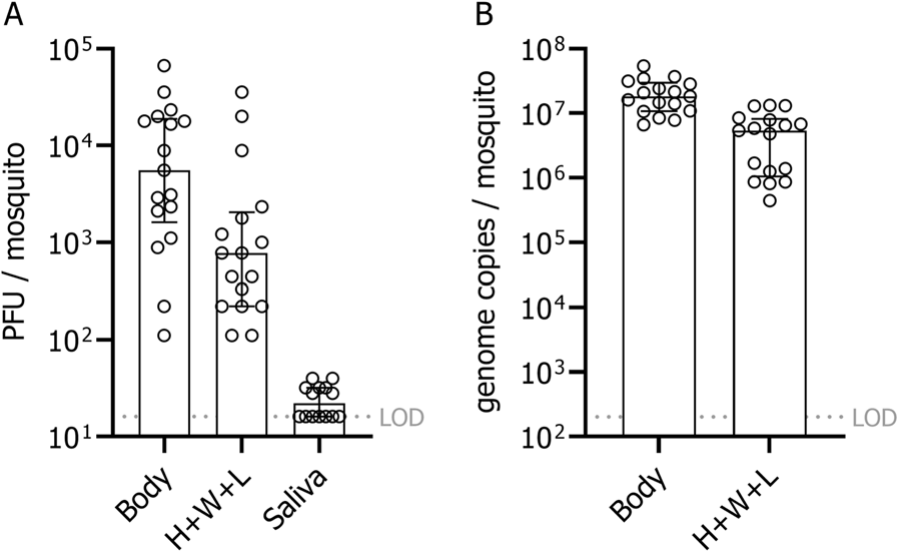
Infectious titres and viral genome copies in *Culex modestus* infected with USUV. **A** Plaque-forming units (PFU) in the mosquito body; head, wings and legs (H + W + L)M; and saliva samples. **B** Viral RNA genome copies in the mosquito body and the head, wings and legs (H + W + L). The bars represent the median with the interquartile range. *LOD* limit of detection

Most *Culex modestus* (60.7%) had a positive USUV infection in the abdomen with complete (100%) dissemination to the heads, wings and legs (Fig. 1). Of the mosquitoes with a disseminated infection, almost all had detectable, infectious USUV in their saliva (88.2%). Overall, the transmission efficiency of this *Culex modestus* population was 53.6%. These mosquitoes had a median USUV infectious titre of 5560 PFU/body [95% confidence interval (CI) 2110–17,800]; 778 PFU (95% CI 222–1780) in the head, wings, and legs; and 22 PFU (95% CI 16–32) in the saliva [Fig. 2A]. The bodies and the heads, wings and legs had a median titre of 1.84 × 10^7^ (95% CI 1.09–2.82 × 10^7^) and 5.43 × 10^6^ (95% CI 1.25–7.94 × 10^6^) viral genome copies, respectively (Fig. 2B). In our previous study, we observed a similar infection rate (60%, *n* = 3/5) but lower dissemination and transmission rates (dissemination: 66.7%, *n* = 2/3; transmission: 50%, *n* = 1/2) in *Culex modestus* experimentally infected with USUV-spiked chicken blood [3]. Although it is unclear how third-generation lab-reared mosquitoes differ from the field-collected, these results clearly indicate that *Culex modestus* from Belgium are capable of transmitting USUV.

Similar to *Culex pipiens* form *pipiens*, *Culex modestus* are widespread across Europe, feed interchangeably between birds and humans, and can outlast the cold season by overwintering (reviewed elsewhere [7]). *Culex modestus* are considered primary vectors for West Nile virus in southern Europe [8–10] and potential vectors of parasitic heartworms (*Dirofilaria immitis*) [11], avian malaria (*Plasmodium relictum*) [12] and trypanosomes [13]. While eco-epidemiological data are needed to incriminate this species as a vector of USUV, this study using a third-generation (F_3_) Belgian field colony, our previous vector competence study using wild-type (F_0_) Belgian mosquitoes, and surveillance data from the Czech Republic [14, 15] indicate that *Culex modestus* are likely vectors of USUV.

Given the increasing evidence that *Culex modestus* is an important vector for human and animal pathogens, there have been few studies conducted on this species. A quick PubMed search yields only 122 results for “*Culex modestus*” from 1964 to June 2024. In comparison, these results are less than 4% of those obtained when searching “*Culex pipiens*” (3435 results as of June 2024). Future research on the transmission of USUV and other mosquito-transmitted pathogens in Europe should take *Culex modestus* into consideration.

## Data Availability

The datasets generated and/or analysed during the current study are available in The Open Science Framework repository (10.17605/OSF.IO/QH4GA).

## Abbreviations

ATP: Adenosine triphosphate
BHK: Baby hamster kidney
CI: Confidence interval
DR: Dissemination rate
FBS: Foetal bovine serum
H + W + L: Head, wings, and legs
IR: Infection rate
PBS: Phosphate-buffered saline
PFU: Plaque-forming unit
qRT-PCR: Quantitative real-time reverse-transcription polymerase chain reaction
RH: Relative humidity
s.l.: Sensu lato
TCID_50_: 50% tissue culture infectious dose
TE: Transmission efficiency
TR: Transmission rate
USUV: Usutu virus
